# Testosterone treatment improves liver function and reduces cardiovascular risk: A long-term prospective study

**DOI:** 10.1080/2090598X.2021.1959261

**Published:** 2021-08-09

**Authors:** Ahmad Al-Qudimat, Raed M. Al-Zoubi, Aksam A. Yassin, Mustafa Alwani, Omar M. Aboumarzouk, Khaled AlRumaihi, Raidh Talib, Abdulla Al Ansari

**Affiliations:** a Surgical Research Section, Department of Surgery, Hamad Medical Corporation, Doha, Qatar; b Department of Chemistry, Jordan University of Science and Technology, Irbid, Jordan; c Department of Surgery, Division of Urology/Andrology, Hamad Medical Corporation, Doha, Qatar; d Center of Medicine and Health Sciences, Dresden International University, Dresden, Germany; e School of Medicine, Jordan University of Science and Technology, Irbid, Jordan

**Keywords:** Testosterone therapy, renal function, men, hypogonadism, cardiovascular risk factor, mortality

## Abstract

**Objectives**: To report the association between testosterone treatment in hypogonadal men with hepatic steatosis, non-alcoholic fatty liver disease and cardiovascular disease (CVD). **Methods:** A prospective study was conducted to assess the physiological and functional performance of the long-term effects of testosterone undecanoate treatment on hepatic steatosis in 496 hypogonadal men. Two groups were studied, the treatment group (T-group) of 312 patients treated with TU 1000 mg every 12 weeks and followed for 8 years, and an untreated control group (C-group) of 184 patients. We evaluated liver functions and Fatty Liver Index (FLI) according to Mayo Clinic parameters and guidelines. **Results:** The T-group showed a decrease in the FLI (from a mean [SD] of 83.70 [12.15] to 67.12 [19.21]), bilirubin (from a mean [SD] of 1.69 [4.21] to 1.31 [1.91] mg/dL), triglycerides (from a mean [SD] of 254.87 [92.99] to 213.37 [66.91] mg/dL), and gamma-glutamyl-transferase (from a mean [SD] of 39.45 [11.51] to 29.11 [7.68] U/L) over the duration of the study. Other parameters were also reduced in the T-group such as body mass index (from a mean [SD] of 31.59 [4.51] to 29.50 [3.84] kg/m^2^) and waist circumference (from a mean [SD] of 107.51 [9.95] to 101.86 [9.28] cm). A total of 25 deaths (7.8%) were recorded in the T-group, among them, 11 (44%) were related to CVD. While in the C-group 28 deaths (15.2%) were recorded and all the reported deaths (100%) were related to CVD. **Conclusions:** The findings suggest that long-term testosterone therapy in hypogonadal men improves liver function. While, the physiological and functional improvements in the liver may be associated with a decrease in CVD-related mortality.

Abbreviations

ALT: alanine transaminase; AR: androgen receptor; AST: aspartate transaminase; BMI: body mass index; CVD: cardiovascular disease; FLI: Fatty Liver Index; γ-GT: gamma-glutamyl-transferase; MetS: metabolic syndrome; LDL: low-density lipoprotein; NAFLD: non-alcoholic fatty liver disease; RCT: randomised controlled trial; T2DM: type II diabetes mellitus; TT: total testosterone; TTh: testosterone therapy; TU: testosterone undecanoate; WC: waist circumference

## Introduction

The prevalence of hepatic steatosis has substantially increased in the last decade and reached 10–24% worldwide, with a strong association with type II diabetes mellitus (T2DM) and obesity according to several studies, especially in Western societies [1].

The main characteristic of hepatic steatosis is the fat deposition in liver and is referred to as non-alcoholic fatty liver disease (NAFLD). NAFLD is a liver inflammation that can lead to cirrhosis, liver failure, and often hepatocarcinoma [2]. Hepatic steatosis is also associated with an increase incidence of metabolic syndrome (MetS), T2DM, and obesity, where the key components of these diseases include hyperinsulinaemia, dyslipidaemia, and insulin resistance [3,4]. The prevalence of NAFLD ranges from 50% to 75% in patients with T2DM and in obese patients from 80% to 90% [5–11].

Hepatic steatosis contributes to the increase in the risk of cardiovascular events amongst patients with T2DM (odds ratio 1.84) [12–15]. Moreover, the aetiology of hepatic steatosis and atherosclerosis are usually considered tissue-specific manifestations of the same cardiometabolic pathology [16]. MetS and T2DM are associated with low testosterone and considered factors for cardiovascular risk [17–19]. T2DM is ubiquitous in men associated with low testosterone levels and known to increase the risk of cardiovascular disease (CVD) [20,21].

Testosterone therapy (TTh) is the primary mainstay treatment for functional hypogonadism, in which it causes many symptoms such as impaired libido, erectile dysfunction, reduced quality of life, fatigue, and depression [22]. Several studies have reported that TTh reduces the risk of dyslipidaemia, insulin resistance, improves glycaemic control and central obesity, and hence contributes to minimising the cardiovascular risk in hypogonadal men [22–31].

Although several reports on TTh have been published, the relationship between hepatic steatosis and testosterone is still limited. A few studies have reported a correlation between hepatic steatosis and low circulating serum testosterone levels [32–35], while others have shown no associations [36].

Völzke et al. [32] reported a retrospective study in men and showed a direct correlation between hepatic steatosis and a total testosterone (TT) level of ≤14.2 nmol/L, after excluding all confounding factors such as age, smoking, T2DM, body mass index (BMI), and visceral adipose tissue. Another study by Kim et al. [33] in healthy Korean men showed that low TT levels were inversely correlated with NAFLD after controlling for the effect of insulin resistance.

Supporting these clinical findings, the beneficial effects of testosterone on hepatic steatosis have been supported by numerous animal studies, as there is an elevation in hepatic lipid deposition in animal models with low testosterone levels [37–40]. TTh exhibited improvement of hepatic physiology on a molecular basis by altering expression of important hepatic regulatory genes [38]. It is thought that TTh improves steatohepatitis and hepatic steatosis by inhibiting macrovesicular lipid droplet formation, suppressing endoplasmic reticulum stress, and promoting very-low-density lipoprotein (LDL) export in castrated male rats [40].

Although clinical and experimental trails recommend a physiological level of testosterone to prevent hepatic steatosis in males, only a few studies have been published on the direct effects of long-term TTh on hepatic steatosis.

In the present study, we assessed the effect of long-term TTh on hepatic pathophysiology, by investigating hepatic steatosis and parameters of liver function in hypogonadal men over an 8-year period.

## Patients and methods

In this prospective observational study, 496 older men (mean [SD] age 59 [9.5] years) were selected. The inclusion criteria were any symptomatic hypogonadal men with a testosterone level of ≤12.1 nmol/L. The exclusion criteria were patients who refused treatment or those with an absolute contraindication for testosterone treatment, e.g. active prostate cancer, male breast cancer, and polycythaemia.

**Figure 1.**
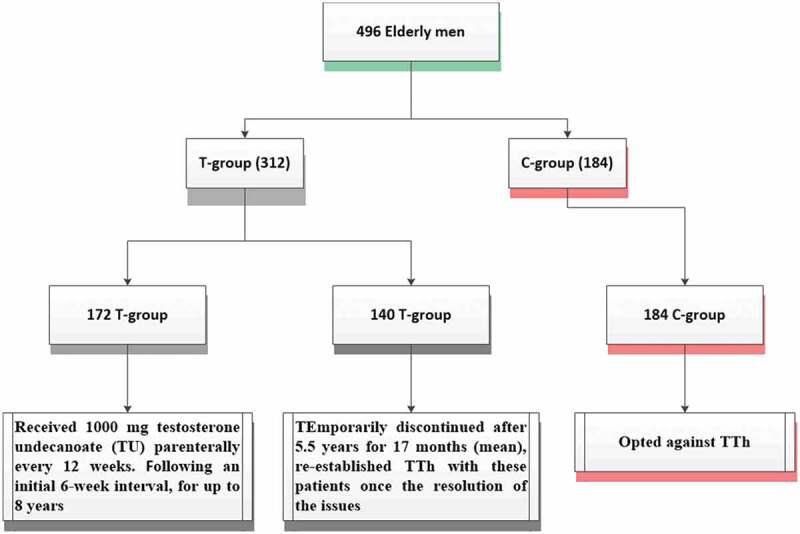
Flow-chart of patient’s recruitment (T-group and C-group)

Study settings were the Institute of Urology and Andrology, Segeberger Kliniken, Norderstedt-Hamburg, Germany, and the Men’s Health Department, Hamad Medical Cooperation, Doha, Qatar. The data collection started in 2004 November until January 2015. A total of 312 men were enrolled in the treatment group (T-group) and received 1000 mg testosterone undecanoate (TU) every 12 weeks, followed for 6 weeks and for up to 8 years (Figure 1). Among them, 140 men had discontinued treatment for 17 months after 5.5 years for reimbursement issues. The remaining 184 patients were selected for the control group (C-group). Both groups had similar baseline characteristics.

Every 6 months, we assessed the effect of long-term TU on hepatic steatosis by measuring the Fatty Liver Index (FLI). The FLI is a well-developed formula published by Bedogni et al. [41]. It is used for identifying and predicting hepatic steatosis by including the following parameters: triglyceride, gamma-glutamyl-transferase (γ-GT), BMI, and waist circumference (WC). All of them summed algorithmically in the following formula:

$$FLI\;=\;\left(\begin{array}{c} e\;0.953*loge\;\left(triglycerides\right)\;\\ +\;0.139*BMI\;+\;0.\\ 718*loge\;\left(ggt\right)\;+\;0.053*WC\;-\;15.745 \end{array}\right)\;\text{\textbackslash{}break}/\;\left(\begin{array}{c} 1\;+\;e\;0.953*loge\;\left(triglycerides\right)\;\\ +\;0.139*BMI\;+\;0.718*loge\;\left(ggt\right)\;\\ +\;0.053*WC\;-\;15.745 \end{array}\right)*100$$

Considering a FLI of <30 and ≥60 to rule out and rule in hepatic steatosis, respectively.

Other parameters of liver function, aspartate transaminase (AST), serum γ-GT, and alanine transaminase (ALT) were enzymatically evaluated using Abbott Alinity-C analytical system (Abbott Laboratories, Lake Bluff, IL, USA). A photometric colour test was used to access total bilirubin (Alinity C-Module; Abbott Laboratories).

The present study adhered to the ethical guidelines of the Declaration of Helsinki (1975) in addition to the approval by the German Ärztekammer (German Medical Association). All patients signed an informed consent before enrolling in the study.

### Statistical analysis

Statistical analysis was performed using the Statistical Package for the Social Sciences (SPSS®) version 12, (Windows package, IBM Corp., Armonk, NY, USA USA) and GraphPad Prism version 8.4.3 (GraphPad Software, La Jolla, CA, USA). Data were expressed as mean (± SD) or as a simple number. Clinical parameters were studied between both groups over the treatment period by linear mixed effects, repeated-measures model with period, group, and their interaction as fixed effects. Data were compared using the chi-squared test or Fisher’s exact test as appropriate. Correlations were performed using Spearman’s correlation coefficient. Regression was used for multivariate test analysis and a *P* < 0.05 was considered statistically significant.

## Results

The characteristics of patients included in this study are described in Table 1. Significant differences were found between the T-group and C-group for BMI (*P* < 0.001), FLI (*P* < 0.001), triglycerides (*P* < 0.05), bilirubin (*P* < 0.05), and WC (*P* < 0.001). The mean (SD) age of the 312 patients in the T-group was 59 (9.5) years, whereas in 184 patients in the C-group it was 66.1 (7.6) years.

**Table 1.** Baseline characteristics of the T-group and C-group

Characteristic	T-group	C-group	*P*
*N*	312	184	
Mean (SD):			
Age, years	59 (9.5)	66.1 (7.6)	
Testosterone level, nmol/L	7.81 (2.32)	9.22 (3.41)	<0.001
WC, cm	107.51 (9.95)	100.76 (9.54)	<0.001
Weight, kg	98 (12)	92 (9)	NS
BMI, kg/m^2^	31.54 (4.51)	29.50 (3.31)	<0.001
FLI	83.70 (12.15)	68.81 (19.27)	<0.001

NS, not statistically significant.

**Figure 2.**
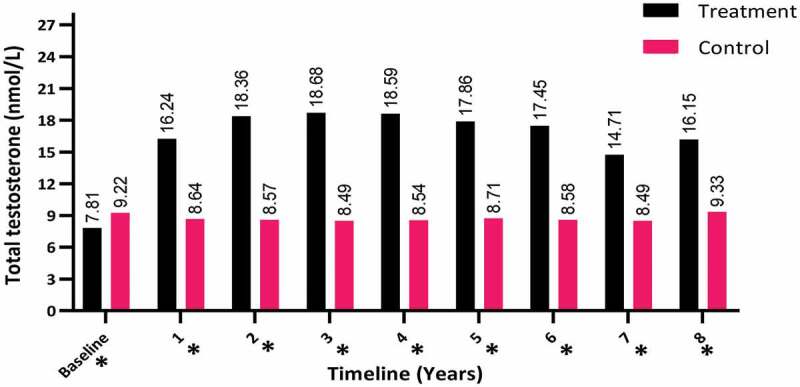
The TT levels at baseline and over the 8-year period for the 312 hypogonadal men in the T-group and 184 untreated hypogonadal men in the C-group (*P < 0.001)

The TT levels increased in the hypogonadal men with TTh (T-group) during the first year of follow-up (from a mean [SD] of 7.81 [2.32] to 16.15 [1.98] nmol/L; *P* < 0.001) and compared to the C-group with a mean (SD) of 9.22 (3.41) to 9.33 (2.87) nmol/L, as shown in Figure 2.

**Figure 3.**
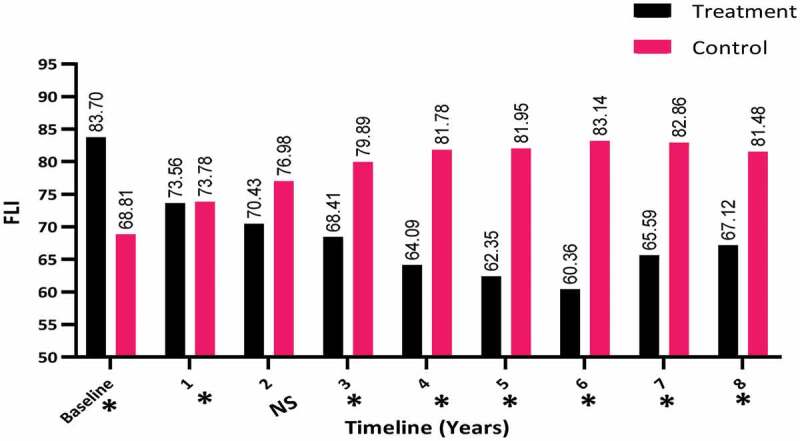
The FLI at baseline and over the 8-year period for the 312 hypogonadal men in the T-group and 184 untreated hypogonadal men in the C-group. *significant; N.S., not significant

The change in the FLI was also investigated between the two groups over the 8-year period. There was a decrease in the FLI in the T-group (from a mean [SD] of 83.70 [12.15] to 67.12 [19.21]) compared to the C-group, in which there was a clear increase in the FLI from a mean (SD) of 68.81 (19.27) to 81.48 (16.84), which was significant (*P* < 0.001) especially for years 3–8 (*P* < 0.001), as shown in Figure 3. There was no significant difference between the two groups in the second year.

**Figure 4.**
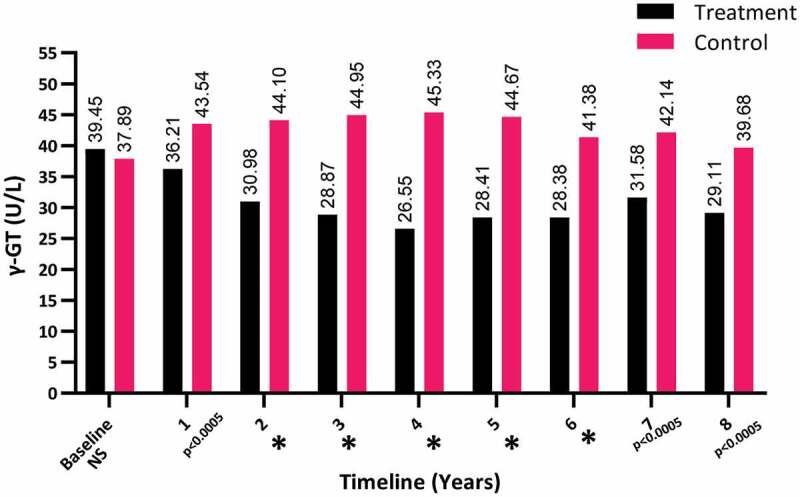
The γ-GT levels at baseline and over the 8-year period for the 312 hypogonadal men in the T-group and 184 untreated hypogonadal men in the C-group (*P < 0.001). N.S., not significant

The γ-GT levels dramatically dropped over the 8-year period in the T-group from a mean (SD) of 39.45 (11.51) to 29.11 (7.68) U/L (*P* < 0.001), whereas there was a steady increase in the γ-GT levels in the C-group, from a mean (SD) of 37.89 (29.61) to 39.68 (26.821) U/L (*P* < 0.05), as shown in Figure 4.

**Figure 5.**
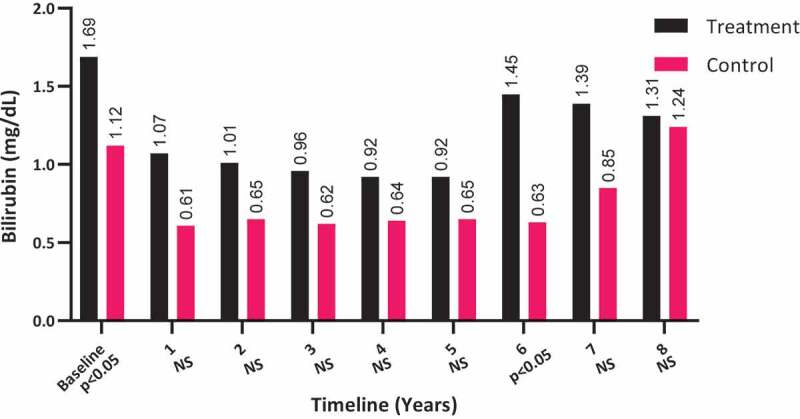
The bilirubin levels at baseline and over the 8-year period for the 312 hypogonadal men in the T-group and 184 untreated hypogonadal men in the C-group. N.S., not significant

Bilirubin levels did not significantly change among the T-group patients (from a mean [SD] of 1.69 [4.21] to 1.31 [1.91] mg/dL; *P* = 0.172) and remained unmoved among the C-group patients (from a mean [SD] of 1.12 (7.11) to 1.24 (2.05) mg/dL; *P* > 0.05), as shown in Figure 5. It is worth noting that the bilirubin levels were elevated in T-group at baseline (*P* < 0.05), which were reduced to comparable levels by 8-years compared to the C-group.

**Figure 6.**
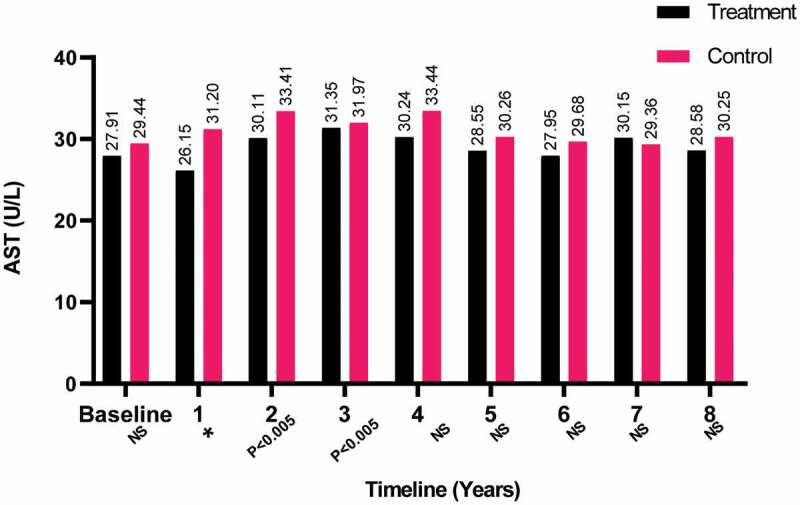
The AST levels at baseline and over the 8-year period for the 312 hypogonadal men in the T-group and 184 untreated hypogonadal men in the C-group (*P < 0.001). N.S., not significant

Furthermore, the AST levels were also found to be steady with baseline over the 8-year period for both groups, as shown in Figure 6. Although several fluctuations that were statistically significant occurred with AST during the study for the T-group. Significant results in AST were found for the C-group in the second and fourth years with *P* < 0.05 and *P* < 0.05, respectively.

**Figure 7.**
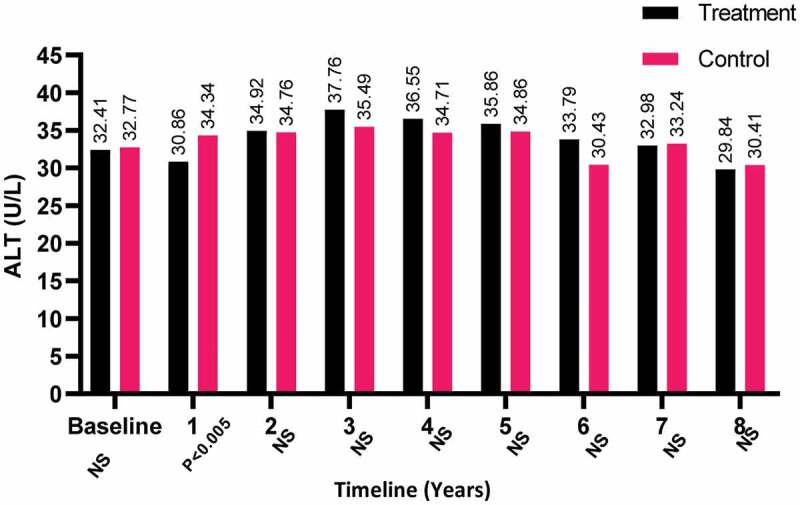
The ALT levels at baseline and over the 8-year period for the 312 hypogonadal men in the T-group and 184 untreated hypogonadal men in the C-group. N.S., not significant

The ALT levels declined slightly for both patient-groups but not significantly by the end of the study period, whereas levels increased in the T-group compared to the C-group in the third year and was found to be statistically significant in the first year of treatment (*P* < 0.05; Figure 7).

**Figure 8.**
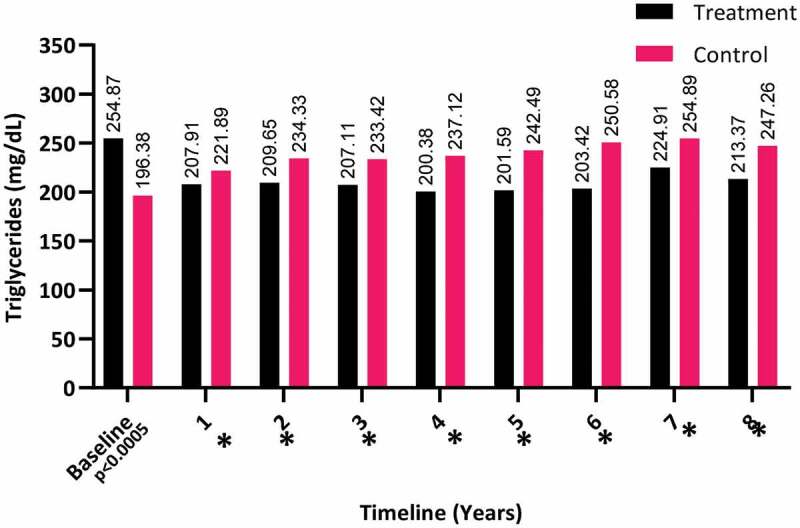
The triglyceride levels at baseline and over the 8-year period for the 312 hypogonadal men in the T-group and 184 untreated hypogonadal men in the C-group (*P < 0.001)

The triglyceride levels showed a decrease in the T-group over the 8-years (from a mean [SD] of 254.87 [92.99] to 213.37 [66.91] mg/dL), whereas it clearly increased in the C-group (from a mean [SD] of 196.38 [92.31] to 247.26 [62.39] mg/dL), as shown in Figure 8. Even though triglycerides continued to decline after the first year and up to 8 years. It continued to show a significant difference between both groups and up to 8 years (*P* < 0.001).

**Figure 9.**
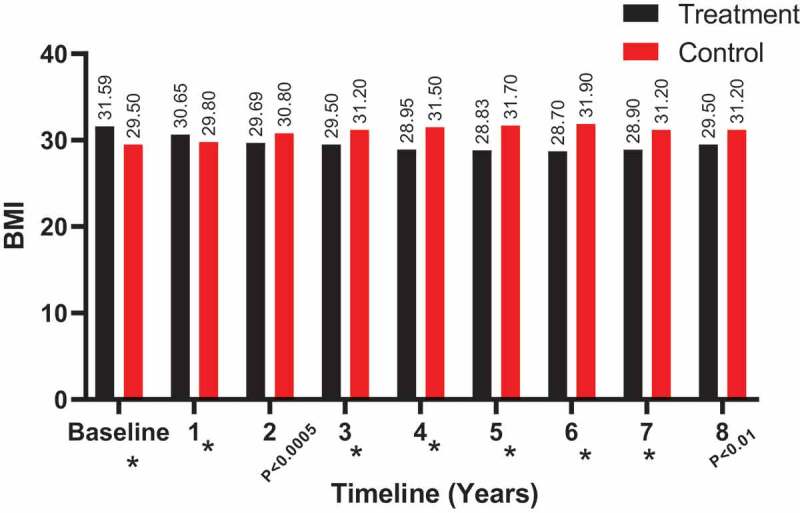
The BMI (kg/m^2^) at baseline and over the 8-year period for the 312 hypogonadal men in the T-group and 184 untreated hypogonadal men in the C-group (*P < 0.001)

The BMI also showed a decrease in the T-group at 8 years (from a mean [SD] of 31.59 [4.51] to 29.50 [3.84] kg/m^2^), whereas it clearly increased in the C-group (from a mean [SD] of 29.50 [3.31] to 31.20 [4.12] kg/m^2^), as shown in Figure 9. Significant results for BMI between both groups were observed from 3 to 8 years.

**Figure 10.**
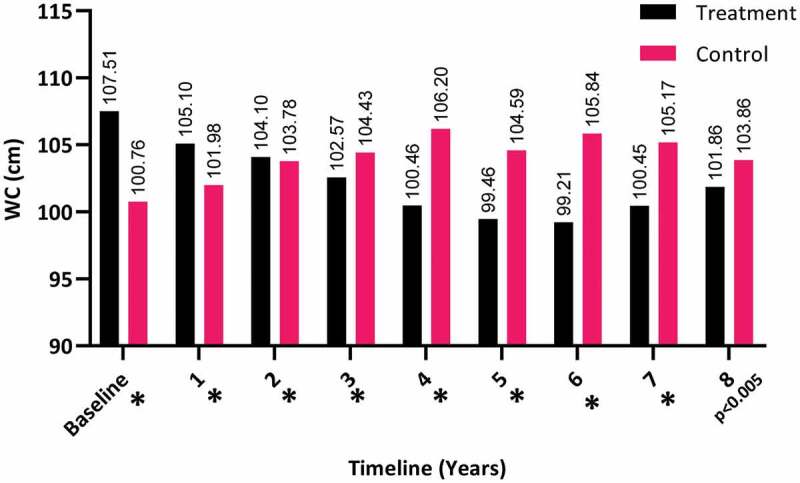
The WC at baseline and over the 8-year period for the 312 hypogonadal men in the T-group and 184 untreated hypogonadal men in the C-group (*P < 0.001)

The WC dropped significantly over the 8 years for the T-group (from a mean [SD] of 107.51 [9.95] to 101.86 [9.28] cm), whereas there was a steady increase in the C-group (from a mean [SD] of 100.76 [9.54] to 103.86 [8.38] cm; *P* < 0.05), as shown in Figure 10.

Furthermore, 28 deaths were recorded for the C-group (14.7%) compared to a lesser rate for the T-group (25 deaths, 7.8%, *P* = 0.035), in which most of the reported deaths from both groups were attributed to CVD (Table 2).

**Table 2.** Comparison of adverse events between the T-group following long-term treatment with TU and the C-group

Adverse events	T-group	C-group	*P*
*N*	312	184	
Deaths, *n* (%)	25 (7.8)	28 (15.2)	0.035
Deaths due to CVD, *n* (%) Myocardial infarction Stroke Heart failure Aortic aneurysm Lung embolism	11 (44)* 5 (20) 2 (8) 2 (8) 1 (4) 1 (4)	28 (100) 13 (48) 7 (27) 3 (11) 2 (7) 2 (7)	

*P < 0.001.

## Discussion

Low testosterone levels in ageing men and the subsequent hypogonadism are associated with different complications such as MetS and the risk of CVD [3,4,42–44]. Additionally, liver steatosis is known to be symptomatic of metabolic dysfunction and could be considered as a hepatic indicator of MetS. Hepatic lipid deposition is independently associated with the low levels of testosterone in men [34]. Even though TTh improves CVD risk, and MetS and T2DM are well-documented [45–49], the long-term testosterone effects on hepatic steatosis are still limited. In the present study, we conducted an observational prospective study on the long-term effect of TTh on hepatic steatosis and liver function and the association with CVD in hypogonadal men.

Several studies have observed a link between low testosterone and NAFLD in their investigations of hypogonadism and liver function. Barbonetti et al. [34] reported on the association between NAFLD and low testosterone levels in male patients with chronic spinal cord injury. Although, that study showed an independent association between the two variables, patients had increased levels of metabolic as well as lifestyle-related risk factors for NAFLD such as reduced physical activity and higher BMI and triglyceride levels, and increased insulin resistance. The prevalence of NAFLD in men with low testosterone levels in this study was 85%. While this relationship is well-known in men with higher BMIs and increased triglycerides, insulin resistance, and γ-GT levels, the association remained even after adjusting for these confounders.

Only a few reports have studied the association between hepatic steatosis and low serum testosterone concentrations in men. For instance, Völzke et al. [32] published a large-scale, population-based cross-sectional study on the effect of low serum testosterone and high serum dehydroepiandrosterone sulphate levels in hepatic steatosis in men. Another two studies in men with hepatic steatosis showed opposing results, the first one identified a strong association with low serum concentrations of testosterone, while the second study revealed no significant differences in testosterone levels compared to healthy men [35,36].

Most of the published studies investigating TTh in hypogonadism focussed on the association with sexual function restoration and improvements in metabolic parameters such as insulin resistance, glycaemic control, obesity, and lean muscle mass rather than the effect on hepatic steatosis and liver function.

Hoyos et al. [50] reported a study of 18 weeks of TTh in obese men with sleep apnea and showed an improvement in metabolic parameters such as insulin resistance and reduced liver fat, although there was no change in body weight overall. On the other hand, Magnussen et al. [51] recently reported a randomised controlled trial (RCT) of low testosterone levels in elderly men and revealed no effect on hepatic fat content as assessed by MRI or magnetic resonance spectroscopy following 6 months of TTh. Correspondingly, another randomised placebo-controlled trial reported by Dhindsa et al. [52] in 44 hypogonadal men with T2DM revealed no change in hepatic fat content following 6 months of TTh and assessed by MRI. Although these studies were doubtless well done, they had smaller cohorts and short-term treatment. In the present study, we present long-term liver function data of hypogonadal men with 8 years of TTh, with a rapid and continuous reduction in FLI during TTh. The present study suggests that long-term TTh improves liver function and reduces lipid accumulation in the liver up to 8 years.

The improvements in other components such as MetS and T2DM could also be mediated by TTh effects on hepatic steatosis [49,53]. The negative correlations between hepatic steatosis and MetS or T2DM components such as insulin resistance, obesity, and dyslipidaemia were previously reported in the literature and supported our present hypothesis [6,54,55]

Moreover, the improvement in WC and BMI combined with the decrease in triglyceride levels in the T-group suggests an improvement in metabolic parameters compared to the C-group. It is well-documented that TTh reduces WC and BMI in hypogonadal men [56]. This improvement of metabolic parameters undoubtedly shows the beneficial long-term treatment effects of TTh on triglycerides and body composition in hypogonadal men that could improve liver steatosis and reduce cardiometabolic disorders and risk factors.

Evidence of the direct effect of testosterone on liver physiology was also reported in animal models [57–59]. It revealed a greater degree of hepatic steatosis and inflammation in hepatic androgen receptor (AR) knock-out mice [57], 5α-reductase type 1 knock-out mice [58] and testicular feminised mice with non-functional AR and low circulating testosterone levels [38]. Other reports showed a severe androgen deficiency in rodents induced by surgical orchidectomy and associated with hepatic steatosis in male mice and rats fed with a high-fat diet, where TTh was found to reduce the hepatic lipid deposition among these testosterone-deficient rodents [38,39].

The role of TTh in reducing expression of acetyl-CoA carboxylase α (*Acaca*), fatty acid synthase (*Fasn*) and sterol regulatory element-binding transcription factor 1 (*Srebf1*) genes involved in hepatic lipid assembly and secretion is well-established [37,38,59]. It has also been hypothesised that androgens could modulate liver fatty acid β-oxidation and lipid synthesis via regulation of SREBF-1 c, a key regulator of fatty acid synthesis, and its primary target gene stearoyl-CoA desaturase (*SCD1*) that catalyses the rate-limiting step in the synthesis monounsaturated fatty acids [57]. In addition to this hypothesis, orchidectomised rats were treated with dihydrotestosterone and an association was found with lower lipid accumulation by decreasing fatty acid, cholesterol synthesis, and increasing β-oxidation [60]. Nevertheless, these mechanistic have only recently arisen, and the validity of these findings from animal studies and the relationship between low testosterone levels and hepatic steatosis in humans requires further studies assessing biopsy samples in TTh clinical trials.

The reduction γ-GT and bilirubin levels in the T-group improved liver function by enhancing metabolic parameters. γ-GT, a glutathione catalase protein and the major thiol antioxidant, is associated with the presence of hepatic steatosis and an elevation in serum γ-GT is known as a marker of oxidative stress and correlates with the presence of CVD [61]. It plays a major role in LDL oxidation, apoptosis, platelet aggregation, and influences plaque rupture [62,63]. The association between elevated γ-GT with CVD and CVD-related mortality is also supported by many epidemiological studies [63–65].

Additionally, Traish et al. [66] reported the effects of TTh on MetS in 225 hypogonadal men and found a decline in AST and ALT levels over 5 years. We observed in our present study a moderate increase in ALT levels, exceeding those of AST at baseline in both groups, and usually observed in the biochemical pattern in hepatic steatosis. However, we report no significant change for AST and ALT levels following TTh treatment, with levels within the normal range for both groups. Other studies have reported a significant association with increasing risk of CVD in men with elevated AST/ALT ratio [67,68]. Others did not find any association [69] and suggest that γ-GT is the only biomarker associated with CVD [63].

The lower CVD-related deaths in the T-group is supported by other reports [70–73]. It is not certain whether the reduction in CVD-related deaths was due to the reduction in hepatic steatosis and improved liver function. Undeniably, patients having NAFLD have a high risk of evolving CVD, and the improvement of hepatic steatosis decreases the risk of CVD [16]. The improvement in hepatic steatosis enhances metabolic parameters and body composition, possibly resulting in the lower number of deaths in the T-group attributed to CVD.

Even though this observational study is not a RCT and has some limitations such as the direct comparison of testosterone vs non-treatment and the limiting the scope of interpretation, the large patient cohort study, and the long-term follow-up of 8 years provide essential clinical data. It also evaluated the effectiveness and safety of long-term treatment with TU injections and a comparison to an untreated hypogonadal control group.

It is worth mentioning that liver parameters such as FLI, bilirubin, triglycerides, BMI, and WC were significantly higher among the T-group compared to the C-group at baseline. This indicates a selection bias as patients may have pre-existing disease while undergoing treatment.

As mentioned in our methodology, the large number of patients discontinuing TTh during the treatment course and hard to follow-up due to reimbursement issues, limited the group differences in liver function and explained some of the parameters in the T-group particularly, between 6 and 8 years.

## Conclusion

The present study highlights that long-term TTh has beneficial effects on liver function in hypogonadal men with low levels of testosterone. As hepatic steatosis is known as a cardiometabolic risk factor, the present study also highlights the low CVD-related mortality rate with the improvement of liver function. Indeed, placebo-controlled RCTs are still needed to elucidate the impact of TTh on hepatic function and steatosis in association with cardiovascular risk in hypogonadal men.
